# Association between rheumatoid arthritis disease activity, progression of functional limitation and long-term risk of orthopaedic surgery: combined analysis of two prospective cohorts supports EULAR treat to target DAS thresholds

**DOI:** 10.1136/annrheumdis-2015-208669

**Published:** 2016-03-15

**Authors:** Elena Nikiphorou, Sam Norton, Adam Young, Lewis Carpenter, Josh Dixey, David Andrew Walsh, Patrick Kiely

**Affiliations:** 1 Department of Rheumatology, Whittington Hospital NHS Trust, London, UK; 2 Early Rheumatoid Arthritis Study, Department of Rheumatology, St Albans City Hospital, St Albans, UK; 3 Psychology Department, Institute of Psychiatry, Psychology & Neuroscience, King's College London, London, UK; 4 Division of Health & Social Care Research, Faculty of Life and Medical Science, King's College London, London, UK; 5 Centre for Lifespan & Chronic Illness Research, University of Hertfordshire, Hatfield, UK; 6 Department of Rheumatology, New Cross Hospital, Wolverhampton, UK; 7 Arthritis UK Pain Centre, University of Nottingham, Nottingham, UK; 8 Department of Rheumatology, St Georges University Hospitals NHS Foundation Trust, London, UK

**Keywords:** Rheumatoid Arthritis, Disease Activity, Treatment, DMARDs (biologic), DMARDs (synthetic)

## Abstract

**Objectives:**

To examine the association between disease activity in early rheumatoid arthritis (RA), functional limitation and long-term orthopaedic episodes.

**Methods:**

Health Assessment Questionnaire (HAQ) disability scores were collected from two longitudinal early RA inception cohorts in routine care; Early Rheumatoid Arthritis Study and Early Rheumatoid Arthritis Network from 1986 to 2012. The incidence of major and intermediate orthopaedic surgical episodes over 25 years was collected from national data sets. Disease activity was categorised by mean disease activity score (DAS28) annually between years 1 and 5; remission (RDAS≤2.6), low (LDAS>2.6–3.2), low-moderate (LMDAS≥3.2–4.19), high-moderate (HMDAS 4.2–5.1) and high (HDAS>5.1).

**Results:**

Data from 2045 patients were analysed. Patients in RDAS showed no HAQ progression over 5 years, whereas there was a significant relationship between rising DAS28 category and HAQ at 1 year, and the rate of HAQ progression between years 1 and 5. During 27 986 person-years follow-up, 392 intermediate and 591 major surgeries were observed. Compared with the RDAS category, there was a significantly increased cumulative incidence of intermediate surgery in HDAS (OR 2.59 CI 1.49 to 4.52) and HMDAS (OR 1.8 CI 1.05 to 3.11) categories, and for major surgery in HDAS (OR 2.48 CI 1.5 to 4.11), HMDAS (OR 2.16 CI 1.32 to 3.52) and LMDAS (OR 2.07 CI 1.28 to 3.33) categories. There was no significant difference in HAQ progression or orthopaedic episodes between RDAS and LDAS categories.

**Conclusions:**

There is an association between disease activity and both poor function and long-term orthopaedic episodes. This illustrates the far from benign consequences of persistent moderate disease activity, and supports European League Against Rheumatism treat to target recommendations to secure low disease activity or remission in all patients.

## Introduction

Treating rheumatoid arthritis (RA) to target (T2T) has become an internationally agreed standard of good practice^1^ embodying the principle that rapid attainment of remission, or low disease activity, can halt joint damage and maintain good quality of life. European League Against Rheumatism (EULAR) guidelines for the management of RA are predicated on the T2T principle, and recommend use of both conventional synthetic disease modifying antirheumatic drugs (csDMARDs) and biologics DMARD (bDMARD) to achieve this. Some countries and healthcare systems restrict use of bDMARDs to patients with a persistent disease activity score (DAS28) of 5.1 or more,^2^ ^3^ well above the highest suggested T2T DAS28 of 3.2.^1^

We have previously reported a low likelihood of achieving low DAS (LDAS) in patients with RA with a DAS28 score in the moderate range, 3.2–5.1 (mDAS), using csDMARD therapies in a real world setting. In patients with mDAS at year 1, only 27% achieved LDAS at year 2. In those with a year 1 DAS28 of 4.2–5.1, even less achieved LDAS at year 2 and year 3, 16% and 19%, respectively.^4^ The conclusion is that patients with RA with mDAS at year 1 are unlikely to achieve the least demanding T2T standard of LDAS with continued csDMARDs alone. These findings have been supported by other studies with high remission rates observed in patients with moderate disease starting biologics.^5–7^ Similarly, longitudinal relationships between mDAS and functional disability have been reported.^7^ ^8^ However, there remains an important gap in the literature on long-term outcomes of mDAS, particularly surrogate markers of joint destruction such as orthopaedic surgery.

The objectives of this study were to examine associations between disease activity during years 1–5 after first presentation with (i) functional outcome, measured using Health Assessment Questionnaire (HAQ), over the same period, and (ii) orthopaedic interventions over a period of up to 25 years after presentation.

## Methods

### Patient databases

The Early Rheumatoid Arthritis Study (ERAS) is a multicentre inception cohort which recruited 1465 patients with early RA (<2 years disease duration, no prior csDMARD) between 1986 and 1999 from nine hospitals in England, followed yearly for up to 25 years (median follow-up 10 years). The Early RA Network (ERAN) has similar design and recruited 1236 early RA patients (<3 years disease duration) from 23 centres in England, Wales and Ireland between 2002–2012 with a median follow-up of 6 years. Recruitment was based on clinician diagnosis with 70% of patients fulfilling the minimum American Rheumatism Association (ARA) criteria^9^ for RA at baseline and 96% by last visit.

### Clinical, laboratory and functional measures

Clinical, laboratory and functional features, including rheumatoid factor (RF) status, radiographs of hand/feet and treatment, were recorded in both cohorts at baseline, between 3/6 and 12 months, then yearly on standardised case report forms (CRFs).^4^ ^10^ Disease activity was calculated according to the original three variable methods in ERAS (DAS)^11^ ^12^ and the more recent four variable DAS28^12–14^ in ERAN, compatibility achieved with a transformation formula.^15^ HAQ was recorded at every patient visit.^16^ Information on anti-citrullinated peptide antibody (ACPA) positivity was available for a limited number in ERAN only.

### DAS28 severity over time

For the purpose of this study, disease severity over time was defined using the mean of all DAS28 recordings between years 1 and 5. Baseline and 3–6 month assessments were excluded due to the prevalence of non-treatment with csDMARDs at those visits. Nearly all patients had commenced csDMARDs by 1 year (ERAS=95.8%; ERAN=99.8%). Since there are a limited number of observations with equal time intervals, the mean DAS28 over time provides data that are equivalent to the area under the curve method of quantifying dosage. As such, the mean DAS28 can be considered as the average yearly disease activity ‘dose’ while treated. For analysis the mean year 1– 5 DAS28 score for each patient was allocated into one of the following five categories: remission (RDAS≤2.6), low (LDAS>2.6–3.2), low-moderate (LMDAS≥3.2–4.19), high-moderate (HMDAS 4.2–5.1) or high DAS (HDAS>5.1). The mDAS category was split into two levels based on earlier findings in ERAN of differences in outcomes between these groups.^4^

### Treatment profiles

Patients were treated according to usual care in all centres, without specific protocols, T2T or other external influences. In ERAS, csDMARD use was mainly sequential monotherapy.^17^ In ERAN, more frequent and earlier use of combination csDMARD therapies and in a small proportion bDMARDs (<2% by 1 year, <10% by 3 years) were employed.^18^ Median time from symptom onset to first rheumatology outpatient visit was 6 months in both cohorts and time to first csDMARD initiation 1 (ERAN) to 2 (ERAS) months later.

### Orthopaedic data and linkage with national data sets

Orthopaedic data from CRFs and two national data sets were merged as previously described.^19^ Hospital Episode Statistics records inpatient and outpatient orthopaedic interventions undertaken at National Health Service (NHS) hospitals in England. The National Joint Registry records hip, knee and ankle arthroplasty (more recently elbow and shoulder) undertaken in the NHS and independent healthcare sectors.

Orthopaedic interventions were categorised by joint type and procedure:^19^ (1) ‘major’ representing large joint arthroplasty (ie, hips, knees, shoulders and elbows) and surgery to the cervical spine; (2) ‘intermediate’ representing mainly wrist, hand and hind/forefoot reconstructive procedures (eg, arthroplasty, synovectomy and arthrodesis).

### Statistical analysis

Summary statistics were used to describe demographic and baseline data between the mean DAS28 groups. HAQ progression between years 1 and 5 was estimated using linear mixed effects modelling incorporating a within-individual random intercept and a random slope for time. Individual HAQ scores at each assessment between 1 and 5 years were included as outcome variables. Time in years was included as a continuous variable (with random slope) allowing for the interpretation as the linear yearly change in HAQ (annualised progression) between 1 and 5 years. Preliminary analysis confirmed that a linear change over time provided acceptable explanatory fit, compared with a quadratic trend with acceleration of the progression rate. DAS28 category was included as dummy coded variables with an interaction term with time. This allows for the estimation of HAQ at 1 year and the rate of HAQ progression for each DAS28 category, accounting for the repeated measurement of HAQ within individuals.^i^ To protect against confounding, the HAQ progression analysis controlled for age at RA onset, gender, baseline RF, erosions, calendar year of first visit, current treatment using dummy coded variables for csDMARD, bDMARDs and steroid prescription in the previous year. To avoid confounding by orthopaedic surgery, HAQ scores measured at time points following surgery were omitted. SEs were estimated using 1000 bootstrap resamples. Tests for differences in HAQ at 1 year and rate of progression between DAS28 categories were corrected for multiple testing using the Bonferroni method (critical z value 2.87, 5% α). Additional analyses, to further probe the association between DAS28 and HAQ, included mean 1–5 year DAS28 as a continuous variable and, separately, individual DAS28 scores as time-dependent variables.

Time in months to first intermediate or major orthopaedic intervention was estimated using multivariate competing risks regression models with censoring at April 2011 (latest date for linkage to national data sets) with death included as a competing risk. For individuals where linkage to national data sets was not possible (eg, died before 1998) censoring was at last visit. As with the HAQ progression analysis, DAS28 categories were included as dummy coded variables, and the analysis protected against confounding by controlling for age at RA onset, gender and baseline HAQ, RF, erosions and calendar year of first visit.^6^ Again, additional analysis included mean DAS28 as a continuous variable. All analyses were carried out in Stata V.14.0.

## Results

### Disease activity categories

**Figure 1:**
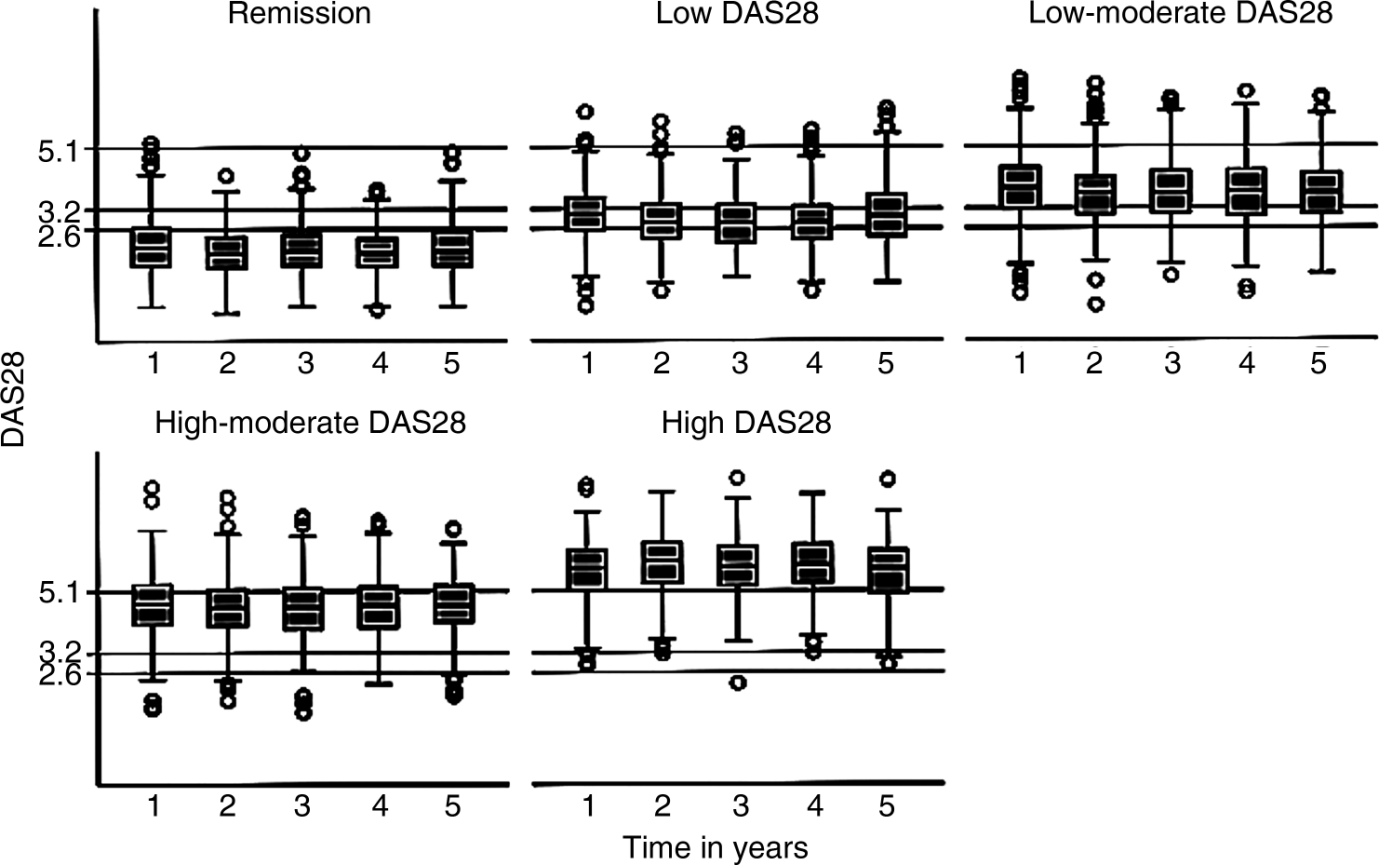
Disease activity score (DAS28) scores from year 1 to 5 by disease activity category.

A total of 2045 (76%) patients had DAS28 recorded at least twice between years 1 and 5. The mean number of DAS28 observations between 1 and 5 years was 3.5 out of 5 possible assessments, and between baseline to 5 years was 5.6 out of 7 possible assessments. Of these, using the mean DAS28 score over time, 21% were in the RDAS category, 15% in the LDAS, 26% in the LMDAS, 21% in the HMDAS and 18% in the HDAS category. The majority of patients were observed to have DAS28 scores within the mean DAS28 category to which they were assigned on the majority of occasions (figure 1). Only 16.4% of patients had more than half of their observations outside the range of their allocated mean DAS28 category, and 6.7% had no observations within their assigned category. The mean within-person SD for DAS28 between years 1 and 5 was 0.84. Table 1 summarises patient demographics, disease measures at the time of recruitment to the ERAS/ERAN cohort, csDMARD use and orthopaedic surgery by DAS28 category. Nearly all patients who were prescribed csDMARDs had started treatment by the 1-year assessment (ERAS=95.8%; ERAN=99.8%).

**Table 1:** Patient demographic, disease measures, csDMARD use and orthopaedic surgery by DAS28 category

	DAS28 categories				
	Remission	Low	Low-moderate	High-moderate	High
Total n (%)	425 (21)	301 (15)	524 (26)	426 (21)	369 (18)
Females (%)	52	57	69	77	82
Age RA onset (mean, SD)	53.4, 14.0	55.5, 13.9	55.8, 13.6	55.7, 14.5	56.5, 13.6
Baseline ESR mm/h (median, IQR)	20, 32	28, 34	33, 43	37, 42	42, 40
Baseline Hb (mean, SD)	13.1, 1.45	13.1, 1.51	12.9, 1.50	12.6, 1.60	12.4, 1.54
Baseline DAS28 (mean, SD)	4.00, 1.42	4.47, 1.27	4.76, 1.21	5.32, 1.16	5.82, 1.05
Baseline HAQ (mean, SD)	0.81, 0.70	0.91, 0.70	1.07, 0.71	1.29, 0.71	1.59, 0.73
Baseline BMI (median, IQR)	25, 5.28	25, 4.87	26, 6.24	26, 6.58	26, 6.20
csDMARDs by 1 year (n, %)	369, 86.8	251, 83.4	471, 89.9	396, 93.0	356, 96.5
csDMARDs weeks to start (median, IQR)	2, 0–6	2, 0–13	2, 0–9	2, 0–6	2, 0–6
bDMARDs by 1 year (n, %)	6, 1.4	1, 0.3	3, 0.6	1, 0.2	4, 1.1

BMI, body mass index; DAS, disease activity score; ESR, erythrocyte sedimentation rate; HAQ, Health Assessment Questionnaire; csDMARDs, conventional synthetic disease modifying antirheumatic drugs; Hb, haemoglobin; RA, rheumatoid arthritis; bDMARDs, biologics DMARD.

### Disease activity and HAQ progression up to year 5

**Figure 2:**
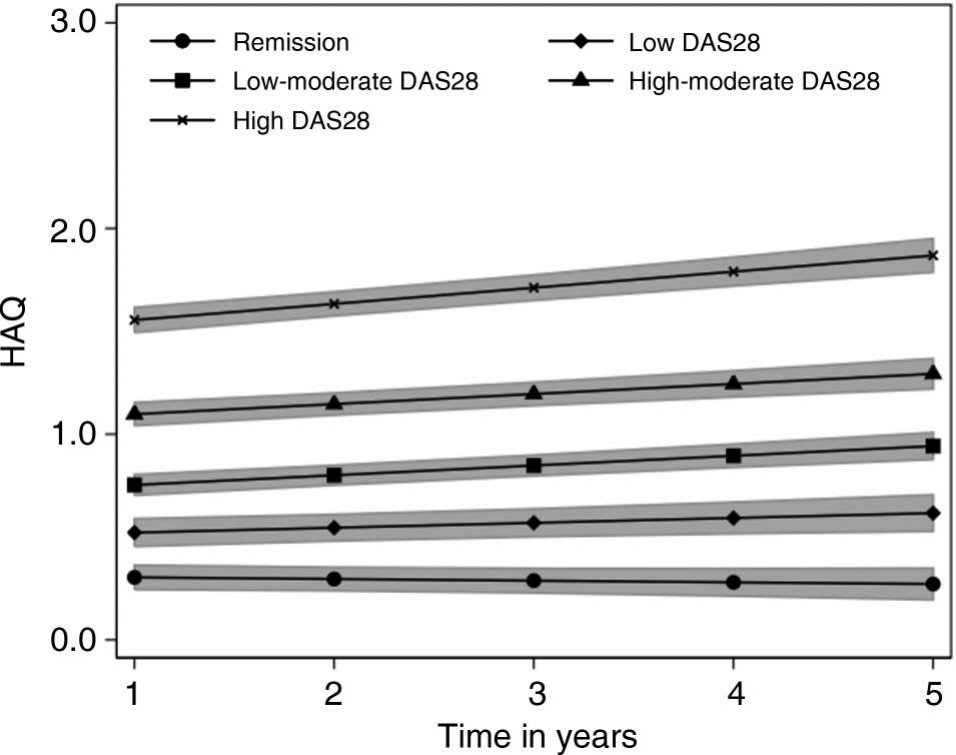
Health Assessment Questionnaire (HAQ) progression by disease activity score (DAS28) category. Shaded areas indicate 95% CIs.

Figure 2 shows the HAQ trajectories for each of the five mean DAS28 categories. There is a clear relationship between DAS28 category and HAQ at year 1 (χ^2^(4)=682.9, p<0.001) and HAQ progression between years 1 and 5 (χ^2^(4)=51.7, p<0.001).

At year 1, HAQ was statistically significantly higher for each DAS28 category compared with the RDAS category and, due to greater progression, was further exaggerated by year 5 (all Bonferroni-corrected contrasts p<0.05). For the remission category HAQ at year 1 was 0.304 (95% CI 0.245 to 0.363) and did not increase significantly with time (annualised progression=−0.008; p=0.345; 95% CI −0.025 to 0.009). The LDAS category had a higher HAQ at year 1 (0.522; 95% CI 0.455 to 0.589) and experienced slow but significant progression over time (0.023; p=0.020; 95% CI 0.004 to 0.044). The LMDAS and HMDAS categories differed in terms of HAQ at year 1 (0.753; 95% CI 0.703 to 0.803 vs 1.097; 95% CI 1.041 to 1.153, respectively) and both progressed significantly and similarly over time (0.047; p<0.001; 95% CI 0.033 to 0.062 and 0.049; p<0.001; 95% CI 0.032 to 0.066, respectively). The HDAS category had the highest HAQ at year 1 (1.555; 95% CI 1.494 to 1.616) and experienced the most rapid rate of progression (0.078; p<0.001; 95% CI 0.060 to 0.097). Compared with the RDAS category HAQ progressed at a significantly faster rate in all other DAS28 categories, though following Bonferroni correction only the rates for the LMDAS, HMDAS and HDAS categories were significant. Compared with the LDAS category only the HDAS category progressed at a significantly faster rate (Bonferroni-corrected contrast p<0.05).

Analysis with mean DAS between 1 and 5 years as a continuous variable indicated that each one unit increase in mean DAS28 score was associated with a 0.193 (95% CI 0.178 to 0.208; p<0.001) higher HAQ score at 1 year, and an annual HAQ progression rate that is increased by 0.005 (95% CI 0.001 to 0.010; p=0.023). Further analysis, including individual DAS scores at each assessment as a time-dependent variable gave similar results, although the impact of DAS28 on annualised HAQ progression was enhanced. Each one unit increase in DAS28 score at the same assessment as HAQ was associated with a 0.176 (95% CI 0.178 to 0.208; p<0.001) higher HAQ score at 1 year, and an annual HAQ progression rate that is increased by 0.013 (95% CI 0.008 to 0.170; p<0.001). Sensitivity analysis (data not shown) indicated the same pattern of association between DAS28 category and HAQ between the ERAS and ERAN cohorts. Within each DAS28 category none of the rates of HAQ progression differed significantly between ERAS and ERAN. A further sensitivity analysis excluded patients with >50% of their scores outside of the DAS28 category to which they were assigned. The estimates of HAQ progression did not differ between DAS28 categories, though CIs were wider due to loss of precision.

### Disease activity and prediction of orthopaedic surgery

**Figure 3:**
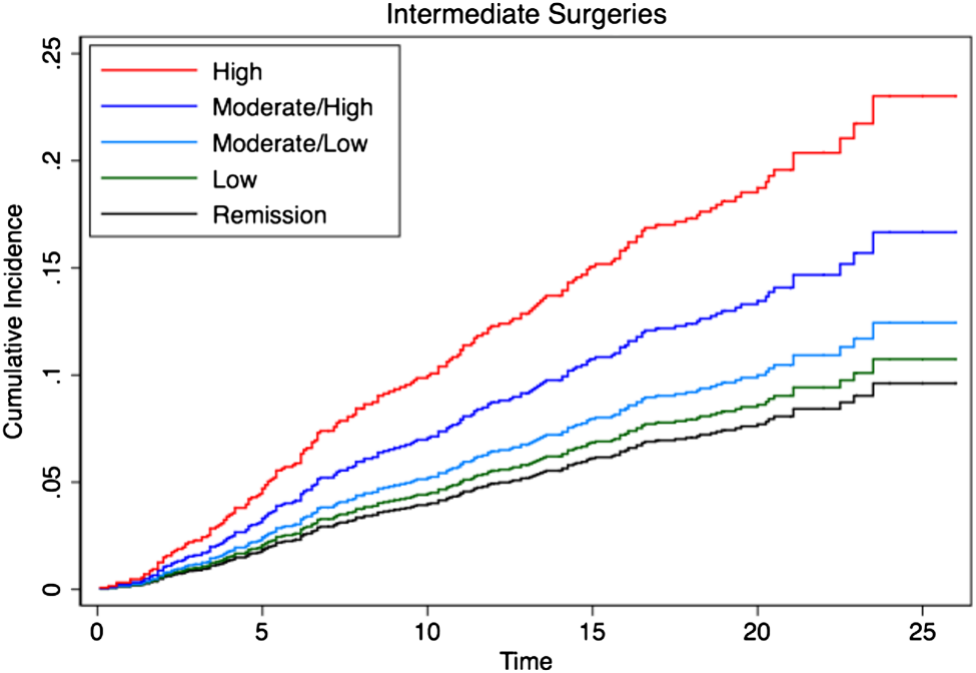
Cumulative incidence for intermediate orthopaedic surgery by disease activity score (DAS28) category over time.

**Figure 4:**
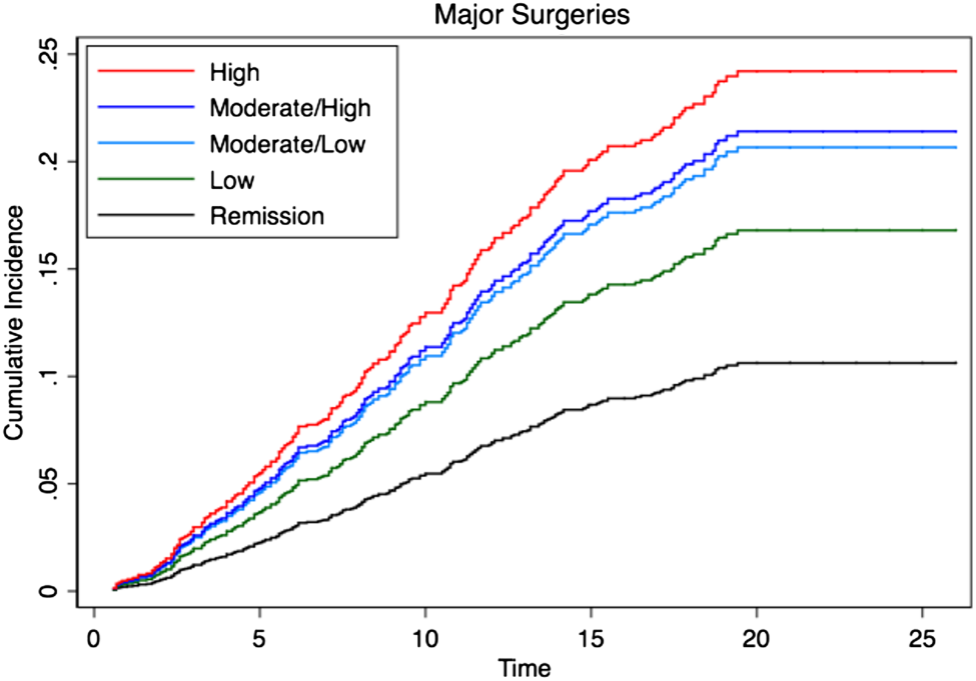
Cumulative incidence for major orthopaedic surgery by disease activity score (DAS28) category over time.

During 27 986 person-years follow-up, a total of 392 intermediate and 591 major surgeries were observed. This translates to a crude incidence rate of 14.0 (95% CI 12.7 to 15.5) per 1000 person-years and 21.1 (95% CI 19.4 to 22.9) per 1000 person-years, respectively. The 10-year cumulative incidence of intermediate surgery was 8.3% (95% CI 7.1% to 9.7%) and major surgery 11.7% (95% CI 10.4% to 13.4%). Figures 3 and 4 show intermediate and major surgery cumulative incidence, respectively, in each of the DAS28 categories up to 25 years estimated from multivariate competing risks regression models. An increasing risk for both intermediate and major surgery was seen moving from low to moderate to high DAS28 categories (table 2).

**Table 2:** HRs (95% CI) for intermediate and major orthopaedic surgery by DAS28 category

	DAS28 category				
	Remission†	Low	Low-moderate	High-moderate	High
Intermediate surgery	1.00	1.13 (0.60 to 2.11)	1.33 (0.77 to 2.29)	1.80* (1.05 to 3.11)	2.59* (1.49 to 4.52)
Major surgery	1.00	1.65 (0.97 to 2.80)	2.07** (1.28 to 3.33)	2.16** (1.32 to 3.52)	2.48** (1.50 to 4.11)

*p<0.001; **p<0.05.

†Reference category against which other DAS28 categories are compared.

DAS, disease activity score.

Compared with patients in the RDAS category, there was a significantly increased cumulative incidence of intermediate surgery observed in patients in the HMDAS (HR 1.8; 95% CI 1.05 to 3.11) and HDAS (HR 2.59; 95% CI 1.49 to 4.52) categories, and for major surgery a significantly increased cumulative incidence in patients in the HDAS (HR 2.48; 95% CI 1.5 to 4.11), HMDAS (HR 2.16; 95% CI 1.32 to 3.52) and LMDAS (HR 2.07; 95% CI 1.28 to –3.33) categories (table 2). Comparing the LDAS with the RDAS category, the HR for major and intermediate surgery was 1.65 and 1.13, respectively, but no statistical significance was reached (p>0.05).

Further analysis including DAS as a continuous, rather than categorical, variable also supported the relationship between disease activity and increased risk of both intermediate (HR 1.31; 95% CI 1.15 to 1.48) and major surgery (HR 1.22; 95% CI 1.10 to 1.36).

A sensitivity analysis was performed with the analysis conducted separately for each cohort (data not shown). The same increasing trend in HR was observed for increasing DAS28 category with no substantive difference in the estimates between cohorts. A further sensitivity analysis excluded patients with >50% of their scores outside of the DAS category to which they were assigned. The HR estimates for each DAS28 category did not differ substantively compared with the original analysis.

## Discussion

This study reports on functional and orthopaedic surgery outcomes in two large RA inception cohorts, both surrogate markers for failed medical management.^20–23^ Our findings demonstrate an association between increasing disease activity, measured by mean DAS28 after csDMARD initiation, and functional limitation, measured by absolute HAQ scores and the rate of progression of HAQ over 5 years. Patients in the RDAS category had no HAQ progression, demonstrating that functional preservation is achievable; as embodied in the T2T principles.^1^ While predictably patients in the HDAS category showed the highest functional limitation and progression, those in the LMDAS and HMDAS categories also demonstrated significantly greater progression than the RDAS category, illustrating that these are not benign states.

The orthopaedic data support these findings, demonstrating that the cumulative incidence of intermediate and major surgery over 25 years is associated with disease activity between years 1 and 5. In particular, patients in the LMDAS and HMDAS categories had a significantly higher prevalence of major surgery compared with those in RDAS and this was also true for intermediate surgery for those in HMDAS, illustrating the far from benign consequences of sustained mDAS and csDMARD therapy between years 1 and 5. These findings highlight the long-term health burden in patients with RA not achieving early and sustained remission.

Our results are supported by data from the Evaluation et Suivi de POlyarthrites Indifférenciées Récentes (ESPOIR) cohort^8^ where patients with early RA and persistent mDAS demonstrated adverse outcomes, compared with those in sustained remission during the first year; evidenced by increased 3-year radiographic progression, increased Health Assessment Questionnaire Disability Index (HAQ-DI) at 3 and 5 years and a fivefold increase in missed workdays over 5 years.

The observations made in our study have critical implications in countries like England and Wales where eligibility criteria to commence bDMARDs are based on DAS28 thresholds which exclude moderate disease.^2^ This applies to as many as 47% of patients in ERAS/ERAN who were categorised in the mDAS range between years 1 and 5, and would not be permitted to bDMARDs by The National Institute for Health and Care Excellence (NICE). In contrast, Swedish data report that half of all first time tumour necrosis factor (TNF) inhibitor starters in 2011 had a DAS28 of <5.2.^24^ Furthermore, data from the Antirheumatic Therapies In Sweden (ARTIS) register demonstrate the cost-effectiveness of bDMARDs in patients with mDAS and HDAS.^25^ Others have demonstrated the clinical benefits of starting bDMARDs earlier and at lower disease activity levels including mDAS.^7^ ^26^ It is therefore evident that bDMARDs are effective when used as part of a T2T strategy.^1^ In contrast, our data reveal the adverse consequences when restrictive healthcare systems deny patients with mDAS such therapies.

Intriguingly, when comparing the LDAS and RDAS categories, similar functional and orthopaedic outcomes were seen. The annual rate of HAQ progression in the general population aged over 50 has been reported to be 0.01.^27^ Our findings for the RDAS (−0.008) and LDAS (0.023) groups between years 1 and 5 are not significantly different from this, nor from each other. In terms of orthopaedic episodes, the differences observed between the LDAS and RDAS categories for intermediate and major surgery were also not statistically significant. This prompts the question, whether in a T2T strategy it is necessary to reduce disease activity as low as remission, or alternatively whether LDAS is sufficient. Our data would seem to support the EULAR recommendation that LDAS is an acceptable target in patients with established disease.^1^

The real-life setting, large patient numbers and long follow-up are strengths that have enabled an analysis of the consequences of a range of disease activity states in the first 5 years on function and orthopaedic episodes up to 25 years later. The linkage with national data sets and high follow-up rates add to the validity and accuracy of data examined. HAQ is recognised as a predictor of key outcomes of disease such as mortality,^28–30^ work disability^31–33^ and healthcare resource utilisation^34^ and is therefore a powerful outcome measure. This analysis controlled for key parameters of disease which could have influenced the results including year of first visit (as an indirect measure of treatment strategies employed at different times), treatment using dummy coded variables for csDMARDs, and bDMARDs, and steroid prescription in the previous year. However, the approach used does not fully account for confounding by indication, therefore it is not possible to make specific causal inferences about the impact of different treatment regimens on outcome.^35^

In our analysis, allocation of patients into one of five DAS28 categories as an indicator of disease severity over time was based on the mean DAS28 score between years 1 and 5. This represents average disease activity per patient but does not imply that patients will have spent all of the study period persistently in the DAS28 category to which they were allocated, nor indeed that there is no fluctuation in DAS28 scores over time. Findings for the mean DAS28 groupings should be considered in terms of the annual ‘dose’ of DAS28, while on treatment. Nevertheless, as only 16.4% of patients (figure 1) had more than half of their observations outside the range of their category, for many the allocated DAS category indicates a relatively persistent state. This reflects the inability of standard care at that time (largely in the absence of bDMARDs), to achieve contemporary T2T outcomes. A limitation is that, though DAS28 and HAQ data were available beyond 5 years for both cohorts, the number of ERAN patients who provided data beyond 5 years was limited due to recent recruitment. Restricting the analysis to the first 5 years, during which the disease progresses from early to established, increases likely generalisability to modern patients treated with csDMARDs. This study illustrates an association between disease activity and both progression of functional limitation and orthopaedic episodes in RA, with incremental differences apparent in all DAS28 categories, compared with RDAS. This supports the selection of a DAS28 score not higher than 3.2 as a T2T outcome, and in our opinion provides a strong argument for maximising treatment interventions when this has not been achieved. Our data may be used to inform guidelines and recommendations for the use of more intensive therapies including bDMARDs in patients with RA with persistent mDAS. We hope this will translate into a more harmonised approach to the management of RA across the globe with fresh imperatives to adopt T2T strategies to achieve remission or LDAS for the majority of patients.

## Footnotes

i In additional post-hoc analyses, RF or erosion status displayed negligible effect modulation.
